# What’s in a Name?

**DOI:** 10.1007/s40037-019-00552-5

**Published:** 2019-12-09

**Authors:** Gisèle Bourgeois-Law

**Affiliations:** Centre for Health Education Scholarship and Island Medical Program, University of British Columbia, Vancouver, Canada

The authors of ‘Guidelines: The do’s, don’ts and don’t knows of remediation in medical education’ have provided us not only with guidelines for those wishing to develop or improve a remediation program in their institution, but also with a summary of what is currently known on the remediation of medical learners [1]. This paper builds on previous work by several of the authors regarding emerging best practices for remediation programs [2, 3]. In addition to incorporating new knowledge on remediation in the 5 years since Kalet and Chou’s seminal book, the present guidelines have been refined to distinguish between systems level and individual level processes. We might perhaps think of this as a variation of ‘think globally, act locally’—‘think systemically, act individually’.

The first challenge in grappling with remediation, as with any wicked problem, is how to define it. The authors define remediation as ‘the act of facilitating a correction for trainees who started out on the journey towards becoming a physician but have moved off course.’ Since no medical trajectory is perfectly straight, we might ask how far off course a learner must deviate before correction is mandated. There are also varying degrees and means of correction; how extensive or formal must these be to be classified as remediation? As an example, a recent study notes that some preceptors consider providing constructive feedback to be ‘remediation’[4]; since every learner benefits from constructive feedback at some point, this suggests that every learner will undergo some degree of remediation. Perhaps then, remediation might be further conceptualized as a continuum of support ranging from feedback to a formal structured experience.

However, the trigger for formal remediation depends not only on the trainee’s precise location on the continuum of needed support but also, as Krzyzaniak et al. have noted [4], on the individual’s response to corrective feedback. Two individuals with the same knowledge or skills gaps might respond quite differently to constructive feedback or offers of support, with one taking feedback to heart and diligently striving to improve, while the other persists in excuses and denial. The decision to mandate remediation would thus depend as much on the individual’s response as on the size of the learning or skills gap. Where then, among the infinite possible combinations of course deviation and response to feedback, could one draw a line beyond which corrective actions constitute remediation?

Remediation, whether conceptualized as a correction for a few or a continuum of support for all, is highly fraught. Chou et al. suggest in the first guideline that remediation be reframed as ‘a special zone of learning, self-improvement, personal development, resilience building, and an opportunity to practice with feedback.’ Were remediation advertised as such, one might even expect eager learners volunteering to participate. But will reframing remediation remove the stigma, or is this yet another area where the hidden curriculum lurks? What is so negative about needing remediation that learners not only fear it but also distance themselves from peers who require it? As a postgraduate dean told us in a recent study: ‘*I think we’re brutal. Frankly, I see it even in medical students and residents. If you start showing any signs of struggle or weakness the first instinct of many physicians is just to want you to be gone’ *[5]. ‘Othering’ our struggling colleagues thus starts early. In such a culture, no matter what we call it or how we frame it, remediation will continue to be problematic.

Medicine has a strong culture of performance, a culture that leads trainees to view feedback meant to be formative as summative and thus threatening [6]. If feedback is threatening, remediation must be catastrophic. In a profession where the goal is not only performance but independent performance,—to be ‘entrusted’ to perform independently—independence in one’s learning is the first expected step towards full professional status. Is it possible that by explicitly encouraging our students and residents to be self-regulated, independent learners we might inadvertently be increasing remediation’s stigma? Remediation, by definition, is a failure of self-regulated learning: the learner must be told what, when and how to learn, and will be observed and/or tested to ensure that the learning has taken place. Reframing remediation as a ‘special zone of learning’, or even as ‘support’ can’t change that underlying connotation, nor will increasing the numbers of learners who are required to undergo the process. It might even, as we have seen in the world of continuing professional development, lead some learners to conflate general support or improvement strategies with remediation and refuse to engage with them.

Perhaps then, in conjunction with moving towards a culture of improvement rather than performance [6], we might also need to move from a learning culture of self-regulation to one of co-regulation. Having been involved both directly and indirectly in the remediation of practising physicians for many years, this commenter would like to suggest that the most dangerous physicians, and the ones most likely to lose their license to practice, are not those who fail to self-regulate their learning or to perform independently. They are, rather, those who refuse to listen to and incorporate feedback, whether from their patients, their colleagues, or their environment. They are those who fail to seek out or accept co-regulation and who persist in viewing themselves as fully autonomous, self-only regulated professionals.

The questions in the paper’s final paragraph highlight that the discussion around remediation is ultimately a discussion around values. It is also ultimately a discussion around culture, since our culture determines our values. It may be time to move beyond a culture of individual self-regulation to one of community co-regulation. Needing input and support from one’s peers and teachers in training, and from the broader community once in practice, should not be a source of shame or lead to stigmatization. The only stigma should lie in the refusal to accept feedback and needed support in order to improve. If we can get to that place, what we call or how we define remediation may ultimately be a moot point.
